# Combined effects of C225 and 125-iodine seed radiation on colorectal cancer cells

**DOI:** 10.1186/1748-717X-8-219

**Published:** 2013-09-23

**Authors:** Jingjia Liu, Hao Wang, Ang Qu, Jinna Li, Yong Zhao, Junjie Wang

**Affiliations:** 1 Cancer Center, Peking University Third Hospital, Beijing, China; 2 Transplantation Biology Research Division, State Key Laboratory of Biomembrane and Membrane Biotechnology, Institute of Zoology, Chinese Academy of Sciences, Beijing, China

**Keywords:** C225, Apoptosis, DNA repair, γ-H2AX, DNA-PK

## Abstract

**Background:**

To characterize the effect of combined treatment of the anti-epidermal growth factor receptor (EGFR) monoclonal antibody C225 and 125-iodine (^125^I) seed radiation in human colorectal cancer.

**Methods:**

We treated LS180 cells with ^125^I continuous low dose rate radiation in the presence and absence of 100 nM C225. The clonogenic capacity, cellular proliferation, cell cycle distribution, apoptosis, and molecular pathways of the cells following the treatments were analyzed in vitro.

**Results:**

The sensitizer enhancement ratio of C225 was approximately 1.4. Treatment with C225 and radiation alone produced significant inhibition of cell growth, but combination therapy produced greater inhibition than either treatment administered alone. C225 increased the radiation-induced apoptosis and the fraction of γ-H2AX foci positive cells at 48 h after treatment. The Akt phosphorylation level was lower in the cells receiving the combination treatment than in the cells treated with radiation or C225 alone.

**Conclusions:**

These findings indicate that C225 sensitizes LS180 cells to ^125^I seed radiation. Growth inhibition is mediated by inducing apoptosis and not cell cycle arrest. Additionally, we confirmed that C225 impairs DNA repair by reducing the cellular level of the DNA-PKcs and Ku70 proteins. Furthermore, the inhibition of Akt signaling activation may be responsible for the C225-mediated radiosensitization.

## Background

Colorectal cancer (CRC) is the third most common lethal disease, and it accounts for approximately one million new cancer cases and approximately 10% of all cancer deaths annually [1]. Preoperative radiochemotherapy administered concomitantly with 5-fluorouracil has become the standard of care in rectal cancer, especially in tumors of the lower and middle rectum [2]. Recently, high dose rate endorectal brachytherapy has emerged as an alternative neoadjuvant treatment for low-lying rectal cancer [3-5]. In our previous study, we found that brachytherapy with low dose 125-iodine (^125^I) seeds could serve as an effective salvage therapy for recurring rectal cancer [6].

Epidermal growth factor receptor (EGFR) is known to be overexpressed in a wide range of cancers, including ovarian, brain, breast, colorectal, kidney, and pancreatic cancers [7]. Multiple lines of evidence indicate that EGFR is an important determinant of radioresponse and has a radioprotective function. Based on current evidence, EGFR-mediated radioprotection can be conceptually divided into three phases: (a) an immediate early phase that involves DNA repair, (b) suppression of DNA damage-induced apoptosis before and after cell cycle arrest, and (c) a tumor repopulation step that offers a proliferative advantage to tumors emerging from radiation-induced cell cycle arrest [8]. Based on the appreciation of the role of EGFR in cancer, several molecularly targeted agents such as gefitinib, erlotinib, and cetuximab (Erbitux, C225) have been developed to inhibit the activity of this receptor. Gefitinib and erlotinib are FDA-approved as single agents for advanced non-small cell lung cancer (NSCLC), and C225 has been approved for the treatment of advanced colon cancer in combination with cisplatin and for head and neck squamous cell carcinoma (HNSCC) in combination with radiation [7].

There are several compelling reasons to explore the efficacy of combined therapy of C225 with continuous low dose rate (CLDR) radiation. C225 commonly produces cytostatic effects that can prevent tumor cell repopulation during the fractionated course of radiation [9]. The short duration of time to deliver CLDR treatment represents a few doubling times for clonogenic cancer cells [10]. Thus, C225 and CLDR treatments administered together may have more cytostatic effects than either treatment alone. If C225 could also produce a cytotoxic effect and radiosensitization, its effect on reducing cell survival of in colorectal cancer cells can be further increased when combined with CLDR.

In the present study, we investigated the role of C225 in modulating the radioresponse of colorectal cancer cells to ^125^I seed continuous low dose rate irradiation (^125^I-CLDR) in vitro. Clonogenic and proliferation assays revealed that C225 enhanced the antitumor effects of ^125^I-CLDR. While dissecting the mechanism underlying this radiosensitization, we observed that C225 administration increased ^125^I-CLDR-induced apoptosis and impaired the repair capacity of cellular DNA, but did not affect ^125^I-CLDR-induced cell cycle arrest. These effects of C225 on irradiation may have been mediated by its inhibition of Akt activation. In this study, we investigated the combined effect of C225 therapy and ^125^I-CLDR in the treatment of colorectal cancer.

## Results

### Sensitization of LS180 cells to radiation from ^125^I seeds by C225

**Figure 1:**
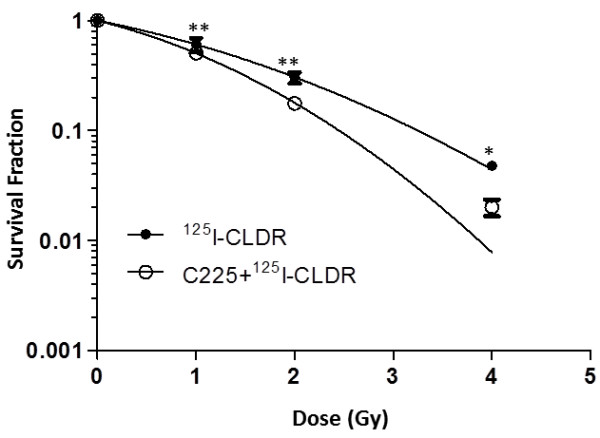
**Effect of C225 on radiation sensitivity.** Cells were exposed to continuous low dose rate radiation from 125-iodine radioactive seeds with and without concurrent treatment of 100 nM of C225. After the treatment, cells were cultured in fresh medium for 14–21 days to assay cell survival with colony formation assay. The dose-survival curves were fitted by the single-hit multitarget model. All data represent two independent experiments, mean ± SD. Two-way ANOVA was used for data analysis. **P < 0.01, *P < 0.05

To assess the radiation-enhancing effects of C225, the cells were exposed to CLDR from ^125^I seeds with and without concurrent treatment with 100 nM of C225. Figure 1 shows the survival curves of in LS180 cells for treatment with radiation alone and in combination with C225. In our study, the survival fraction at 1 Gy, 2 Gy, 4 Gy in 125I-CLDR treated cells was 0.61 ± 0.09, 0.30 ± 0.04, 0.05 ± 0.003, and the corresponding rate in C225 + 125I-CLDR group was 0.51 ± 0.02, 0.18 ± 0.01, 0.02 ± 0.006, respectively. We found there were significant differences concerning clonogenic survival between 125I-CLDR and C225 + 125I-CLDR groups when we used two-way ANOVA for data analysis. The sensitizer enhancement ratio (SER) was approximately 1.4, indicating that C225 increased the radiosensitivity of LS180 cells to radiation from ^125^I seeds. The radiobiological parameters of the LS180 cells are shown in Table 1.

**Table 1:** In vitro radiobiological parameters of LS180 cells (mean ± SD)

	^**125**^**I-CLDR**	**C225 +** ^**125**^**I-CLDR**
D_0_(Gy)	1.20 ± 0.15	0.86 ± 0.03
D_q_(Gy)	0.62 ± 0.30	0.54 ± 0.06
N	1.67 ± 0.31	1.88 ± 0.10
SF_2_	0.30 ± 0.04	0.18 ± 0.01

The values for D_0_, D_q_, and N were determined using the single-hit multitarget model. d = D_0_ (^125^I - CLDR)/D_0_ (C225 + ^125^I-CLDR). D_0_, Mean inactivation dose; D_q_, Quasi-threshold dose; N, extrapolation number; SF_2_, Survival fraction at 2 Gy.

### C225 promotes the radiation-induced inhibition of proliferation

**Figure 2:**
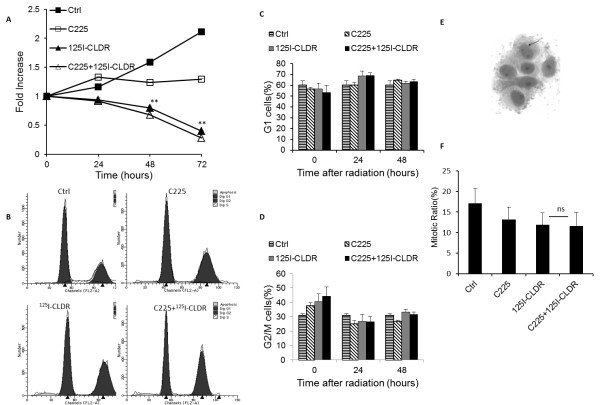
**The antiproliferative effects of C225 and 125-iodine continuous low dose rate radiation. (A)** MTS assay for cellular proliferation at the indicated times after treatment, the exposure dose was 4 Gy (**P < 0.01, compared to C225 + ^125^I-CLDR group, two-way ANOVA). **(B)** Cell cycle distribution at 0 h after treatment, the exposure dose was 4 Gy. **(C, D)** Cell cycle distribution at the indicated times after treatment. **(E)** Morphological analysis after Wright’s-Giemsa staining 48 h after treatment. Arrow indicated a binuclear cell which was a mitotic cell. **(F)** The mitotic ratio at 48 h after treatment, the exposure dose was 4 Gy (ns, P > 0.05, unpaired *t* test was used for data analysis). All data represent three independent experiments, mean ± SD.

We used the MTS proliferation assay to investigate the growth-inhibitory effects of radiation and C225. As shown in Figure 2A, C225 treatment and radiotherapy each significantly inhibited LS180 cell growth when used alone (72 h, Ctrl vs. C225, t = 25.5, P < 0.001; Ctrl vs. ^125^I-CLDR, t = 53.1, P < 0.001), but their combined inhibitory effect was greater than that produced by either treatment alone (72 h, C225 + ^125^I-CLDR vs. C225, t = 31.5, P < 0.001; C225 + ^125^I-CLDR vs. ^125^I-CLDR, t = 3.7, P < 0.01). To further clarify the inhibitory effects of this combination, we detected the cell cycle distribution at indicated times after treatment. G2/M cell cycle arrest only appeared in ^125^I-CLDR and C225 + ^125^I-CLDR treated group (0 h, Ctrl vs. C225, t = 2.4, P > 0.05; Ctrl vs. ^125^I-CLDR, t = 3.5, P < 0.01; Ctrl vs. C225 + ^125^I-CLDR, t = 4.8, P < 0.001), but disappeared at 24 h after treatment (24 h, Ctrl vs. ^125^I-CLDR, t = 1.6, P > 0.05; Ctrl vs. C225 + ^125^I-CLDR, t = 1.7, P > 0.05). G1 cell cycle arrest did not happen in all experiment groups (48 h, Ctrl vs. C225, t = 1.5, P > 0.05; Ctrl vs. ^125^I-CLDR, t = 0.5, P > 0.05; Ctrl vs. C225 + ^125^I-CLDR, t = 1.1, P > 0.05).S cell cycle decreased in three experiment groups within 48 hours after treatment (48 h, Ctrl vs. C225, t = 5.1, P < 0.001; Ctrl vs. ^125^I-CLDR, t = 11.2, P < 0.001; Ctrl vs. C225 + ^125^I-CLDR, t = 11.3, P < 0.001). The cell cycle distribution between C225 + ^125^I-CLDR and ^125^I-CLDR treated cells shew no significant differences within 48 hours after treatment (48 h, C225 + ^125^I-CLDR vs. ^125^I-CLDR, G1, t = 0.6, P > 0.05; S, t = 0.1, P > 0.05; G2/M, t = 0.6, P > 0.05) (Figure 2B, C, D).

We further detected the mitotic ratio at 48 h after treatment in the four groups and found that there were no significant differences (48 h, Ctrl vs. C225, t = 1.2, P = 0.4; Ctrl vs. ^125^I-CLDR, t = 1.6, P = 0.3; Ctrl vs. C225 + ^125^I-CLDR, t = 1.6, P = 0.3; unpaired *t* test) (Figure 2E, F).

### C225 increases radiation-induced cellular apoptosis

**Figure 3:**
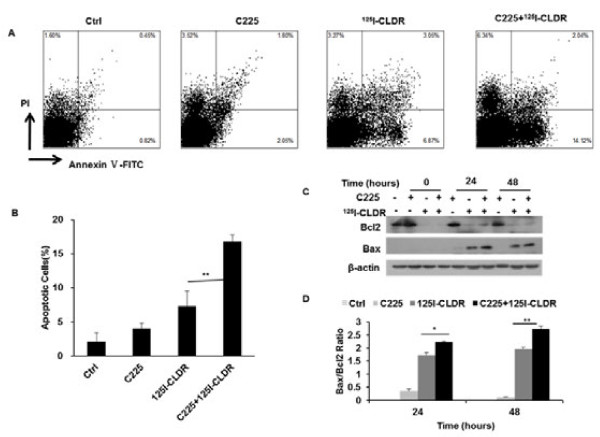
**C225 increases radiation-induced cellular apoptosis. (A, B)** Annexin V-FITC/PI assay was used to detect cellular apoptosis 48 h after treatment, the exposure dose was 4 Gy. **(C, D)** The Bax/Bcl2 ratio was determined by Western blot analysis at the indicated times after the treatment, the exposure dose was 4 Gy. All data represent three independent experiments, mean ± SD. Unpaired *t* test was used for data analysis. *P < 0.05, **P < 0.01 as compared to the ^125^I-CLDR group.

We then detected cell death by annexin V-FITC/PI assay. As shown in Figure 3, both C225 and radiation induced slight cellular apoptosis when administered alone (48 h, Ctrl vs. C225, t = 4.9, P = 0.008; Ctrl vs. ^125^I-CLDR, t = 4.4, P = 0.012; unpaired *t* test), and in the combined treatment, C225 increased radiation-induced apoptosis (48 h, Ctrl vs. C225 + ^125^I-CLDR, t = 24.9, P < 0.001; C225 + ^125^I-CLDR vs. ^125^I-CLDR, t = 6.6, P = 0.003; unpaired *t* test). Furthermore, the Bax/Bcl2 ratio was increased by C225 and radiation either (24 h, Ctrl vs. C225, t = 5.9, P = 0.03; Ctrl vs. ^125^I-CLDR, t = 26.5, P = 0.0014; unpaired *t* test) and increased to highest level by the combined treatment (24 h, Ctrl vs. C225 + ^125^I-CLDR, t = 107.4, P < 0.001; unpaired *t* test). Thus, the combined treatment produced antiproliferative effects by inducing cellular apoptosis as a result of imbalance in the ratio of the pro-apoptotic protein Bax and the pro-survival protein Bcl2.

### C225 reduces the cellular DNA repair capacity

Radiation plays a key role in cancer therapy due to its ability to directly induce DNA damage. In order to determine the cellular DNA damage and repair, immunofluorescence staining was used to determine the nuclear γ-H2AX foci 48 h after treatment. The results revealed a limited number of cells in the control group exhibiting γ-H2AX foci (6.5 ± 0.7%). However, cells receiving combined treatment (59.1 ± 2.2%)demonstrated a significant increase in the γ-H2AX focus-positive cells as compared to those treated with radiation (48.5 ± 0.1%) or C225 (4.5 ± 3.5%) alone. To determine whether DNA repair proteins were expressed, western blotting was performed using lysates from the cells that received the different treatment protocols. The expression levels of DNA-Pkcs (48 h, C225 + ^125^I-CLDR vs. ^125^I-CLDR, t = 5.7, P = 0.005; unpaired *t* test) and Ku70 (48 h, C225 + ^125^I-CLDR vs. ^125^I-CLDR, t = 6.6, P = 0.003; unpaired *t* test) proteins decreased with the combined treatment, suggesting that C225 reduced the cellular DNA repair capacity by reducing the DNA-PKcs and Ku70 protein levels.

### C225 inhibits Akt activation

When the cancer cells overexpressing EGFR were exposed to radiation, the survival and proliferation mechanisms were predominantly activated through signaling via PI3K-Akt and Ras-Erk. Western blot analysis was used to detect the activation of these two pathways. Our results revealed that the phosphorylation level of Akt was lower in the cells receiving the combined treatment (0 h, C225 + ^125^I-CLDR vs. C225, t = 9.2, P < 0.001; C225 + ^125^I-CLDR vs. ^125^I-CLDR, t = 7.3, P = 0.0019; unpaired *t* test) than in those receiving either treatment alone (0 h, Ctrl vs. C225, t = 2.8, P = 0.051; Ctrl vs. ^125^I-CLDR, t = 5.3, P = 0.006; unpaired *t* test). However, there were no significant differences in the activation level of Erk between the different treatment groups.

## Discussion

Preoperative external beam radiotherapy has been shown to increase pathological complete remission and reduce the probability of local recurrence; however, this mode of treatment is also associated with increased risk of therapy-induced side-effects and increased morbidity [3]. High dose rate brachytherapy has been found to be an alternative to external beam radiotherapy in rectal cancer [3,4,11]. Radiation induces an increase in the EGFR expression in cancer cells, and blockade of EGFR signaling sensitizes cells to the effects of radiation [12]. Anti-EGFR monoclonal antibody C225 has been approved for treating HNSCC in combination with radiation because of the synergistic effects of these two treatment approaches [13]. The application of radiosensitizers to sensitize cancer cells to radiation is a promising and emerging strategy. Here, we investigated the capacity of the anti-EGFR mAb C225 to modulate the radiosensitivity and inhibit cellular proliferation in human colorectal cancer cells.

Our results indicated that C225 enhanced the sensitivity of LS180 cells to ^125^I seed radiation (Figure 1). The relatively lower D_0_ value suggested that cancer cells could be killed at reasonably lower doses of radiation when coupled with C225 (Table 1). Previous studies have suggested that the capacity of C225 to modulate cancer cell proliferation and cell cycle distribution might play a central role in its enhancement of radiosensitivity [14]. Our results demonstrated that continuous exposure to C225 inhibited LS180 cell proliferation, and this inhibitory effect was more significant when C225 was combined with radiation (Figure 2A). The growth inhibition of LS180 cells was mediated by apoptosis induction (Figure 3) rather than cell cycle arrest (Figure 2B, C, and D). Moreover, mitotic ratio analysis revealed that combined treatment did not affect the mitotic index (Figure 2E and F).

**Figure 4:**
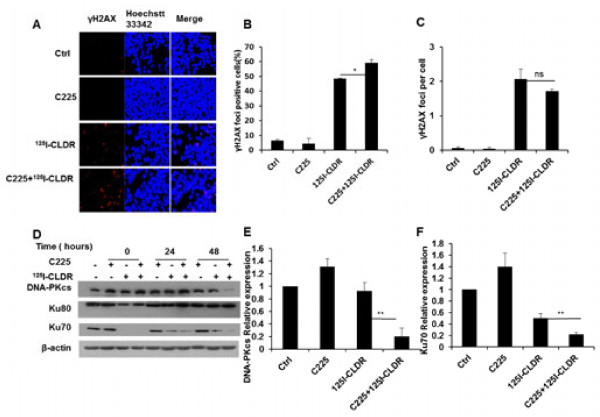
**C225 reduces the cellular DNA repair capacity. (A)** Formation of γ-H2AX foci (red) in LS180 cells 48 h after the treatment; the nuclei were stained with Hoechst 33342 (blue); original magnification, ×20. **(B)** Percentage of γ-H2AX focus-positive cells at 48 h after the treatment. **(C)** Mean number of γ-H2AX foci per cell at 48 h. **(D)** Immunoblotting analysis of DNA-PK(cs), Ku80, Ku70, and actin (loading control) in LS180 cells at the indicated times after the treatment. Representative analysis of DNA-PKcs **(E)** and Ku70 **(F)** protein levels 48 h after the treatment. All data represent three independent experiments, mean ± SD. Unpaired *t* test was used for data analysis. ns (not significant) P > 0.05, *P < 0.05, **P < 0.01 as compared to ^125^I-CLDR group.

We speculate that the enhancement of radiosensitivity by C225 is mediated through inhibiting DNA repair. Among all the radiation-induced damages, DNA double-strand breaks (DSBs) are the greatest threat to cancer cells. In eukaryotic cells, DSBs are repaired primarily by non-homologous end-joining (NHEJ) and homologous recombination (HR) [15]. NHEJ is a faster and more efficient DSB repair pathway than HR [16], and is the dominant mechanism in mammalian cells. The proteins and enzymes of NHEJ include Ku70/80, DNA-PKcs, Artemis, XRCC4, and DNA ligase IV. γ-H2AX foci form at any nascent DSB, and are first visible approximately 3 min post-irradiation by immunoflurorescence microscopy, after which they increase in size until approximately 30 min and then decrease in number with a half-life of several hours [17]. One γ-H2AX focus correlates to one DSB, and one DSB remaining unrepaired in a cell can potentially result in cell death [18]. Recent findings by Banath et al. [19] suggested that the retained γ-H2AX foci at 24 hours after treatment were lethal and those γ-H2AX focus-positive cells would subsequently lose clonogenicity. Our findings showed that the γ-H2AX focus-positive cell fraction was higher in the cells that received the combination therapy than those that received C225 or radiation alone (Figure 4A, B, and C). Furthermore, the lowest levels of DNA-PKcs and Ku70 were found in the cells that received the combination therapy (Figure 4D, E, and F). Therefore, these findings indicate that C225 impairs DNA repair by reducing the cellular level of DNA-PKcs and Ku70.

**Figure 5:**
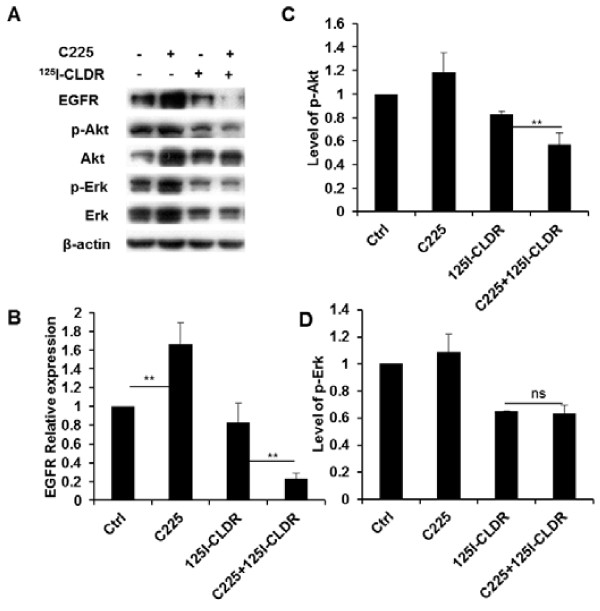
**C225 inhibits the activation of Akt and Erk when combined with radiation. (A)** Immunoblotting of some proteins involving in EGFR signaling pathways at the end of treatment (0 h), the exposure dose was 4 Gy. Cells were lysed immediately after treatment. Representative analysis of the protein levels of EGFR **(B)**, phosphorylated Akt **(C)**, and phosphorylated Erk **(D)**. All data represent three independent experiments, mean ± SD. Unpaired *t* test was used for data analysis.

Furthermore, we determined the biochemical processes responsible for the C225-mediated radiosensitization. An increasing body of data indicates that the PI3K/AKT- and Ras/Raf/MEK/ERK-mediated signaling pathways may govern EGFR-mediated radioresistance [20,21]. In our study, we found that C225 inhibited Akt activation and downregulated EGFR expression when combined with radiation, but did not exert any effects on Erk activation (Figure 5). The inhibition of PI3K-Akt signaling was associated with the downregulation of DNA-PKcs [20]. However, C225-treated cells exhibited EGFR upregulation, suggesting that the cells developed resistance to C225 [22]. Comfortingly, radiation from ^125^I downregulated the expression of EGFR protein, and the cells that received the combination treatment showed lower EGFR protein levels. Thus, C225 played opposite roles in the regulation of EGFR protein in cells treated with or without ^125^I radiation. However, the mechanisms underlying these opposing functions of C225 remain to be elucidated.

## Conclusions

Our findings indicate that C225 sensitizes LS180 cells to ^125^I seed radiation. Growth inhibition was mediated by inducing apoptosis rather than cell cycle arrest. Additionally, we confirmed that C225 impaired DNA repair by reducing the cellular level of DNA-PKcs and Ku70 proteins. Furthermore, C225-mediated radiosensitization may be caused by the inhibition of activation of Akt signaling.

## Materials and methods

### Cell culture

The human colon carcinoma cell line LS180 was kindly provided by the Beijing Institute for Cancer Research. Cells were grown in RPMI-1640 supplemented with 20 mM HEPES (pH 7.4), 100 IU/mL penicillin, 100 mg/mL streptomycin, 4 mM glutamine (Gibco, China), and 10% heat-inactivated fetal bovine serum (FBS; Hangzhou Sijiqing Biological Engineering Materials Company, China) in a humidified atmosphere of 5% CO_2_ at 37°C.

### Radiation with ^125^I radioactive seeds and C225 treatment

We used an in vitro ^125^I seed radiation model that had been developed in our laboratory [23,24]. In this model, the exposure times for delivering doses of 1, 2, 4, 6, 8, and 10 Gy were 36, 73.7, 154.6, 245.8, 345.1, and 460.1 hours, respectively. 100 nM C225 was chosen to treat cells, and the C225 treatment was continuous and concurrent. For example, C225 + ^125^I-CLDR treated cells needs 7 days’ continuous and concurrent C225 treatment when the exposure dose is 4 Gy.

### Antibodies

The human-mouse chimeric anti-EGFR monoclonal antibody (mAb) cetuximab (C225, 5 mg/ml) was provided by ImClone Systems, Inc. (New York, NY, USA). The following primary antibodies were purchased from Cell Signaling Technology (Beverly, MA, USA): rabbit monoclonal anti-γ-H2AX, anti-EGFR, anti-Erk1/2, anti-phospho-Erk1/2, anti-Akt, anti-phospho-Ser473 Akt, anti-Bcl-2, anti-Bax, anti-Ku70, anti-Ku80, and anti-DNA-PKcs antibodies. The following secondary antibodies were obtained for use in this study: Alexa Fluor 546 goat anti-rabbit IgG (H + L) (Invitrogen, Carlsbad, CA, USA) and horseradish peroxidase (HRP)-conjugated anti-rabbit IgG.

### Colony formation assay

After treatment, the cells were trypsinized to generate a single cell suspension, and a specific number of trypan blue-negative cells were seeded into each well of a six-well tissue culture plate. After the plates were incubated for 14–21 days, colonies were stained with Wright’s-Giemsa solution and manually counted. Colonies containing >50 cells were scored, and three replicate dishes containing 10–150 colonies per dish were counted for each treatment.

### Cell proliferation assay

After treatment, the cells were trypsinized to generate a single cell suspension, plated at 5 × 10^3^ cells/well in 96-well plates containing 200 μL growth medium, and allowed to attach for 24 h. At harvest, the medium was removed from the appropriate wells, replaced with 100 μL MTS solution (2.5 mg/mL, Beijing PreGene Biotechnology Company, Ltd. Beijing, China), and incubated for 2 hours at 37°C. After incubation, the plate was analyzed on a plate reader by SoftMax program (Molecular Devices Corp., Menlo Park, CA, USA).

### Cell cycle analysis

The cells were harvested by trypsinization at the indicated times after treatment, washed with ice-cold phosphate-buffered saline (PBS), fixed in 75% ethanol, and stored at −20°C. Prior to analysis, 1 μg/mL propidium iodide (PI) and 1 mg/mL RNAse A were used to label the DNA. Flow cytometry (FACSCalibur, BD Biosciences, San Jose, CA, USA) was used for cell cycle analysis.

### Mitotic ratio analysis

Cells were harvested by trypsinization at 48 h after treatment, treated with ice-cold 0.56% KCl for 3 minutes, pelleted by centrifugation for 10 min at 400 × g, resuspended in Carnoy’s fluid (methanol:glacial acetic acid = 3:1), spread on a slide, air dried, and stained with Wright’s-Giemsa solution. The binuclear cells were observed under a light microscope.

### Apoptotic cell death

The Annexin V-FITC/PI assay (Beijing Zoman Biotechnology Company, Ltd. Beijing, China) was performed according to the manufacturer’s instructions. Briefly, 5 × 10^5^ cells were added to 200 μL of staining solution containing 195 μL of apoptosis buffer and 5 μL Annexin V-FITC. Samples were incubated in the dark at room temperature or 4°C for 30 minutes, after which 300 μL of apoptosis buffer was added. Five minutes before analysis, 10 μL PI was added for chromatin staining. The samples were then analyzed using a Coulter Epics XL cytometer.

### Immunofluorescence staining for γ-H2AX foci

The cells were harvested by trypsinization 48 h after the treatment, treated with ice-cold 0.56% KCl for 3 minutes, and pelleted by centrifugation for 10 min at 800 rpm. The pellets were then resuspended in Carnoy’s fluid (methanol:glacial acetic acid = 3:1), spread on a slide, air dried, and rinsed with 95% ethanol and purified water. Cells were permeabilized in 0.3% Triton X-100 at room temperature for 15 min, and washed three times for 10 min each in PBS. The cells were then blocked with a solution of 1% BSA, 0.3% Triton X-100, and PBS (room temperature) for 2 h, and washed three times for 10 min each in PBS. The samples were then incubated with primary monoclonal anti-γ-H2AX antibody (1:400) in 1% BSA, 0.3% Triton X-100, and PBS overnight at 4°C, washed in PBS three times for 10 min each, and incubated with Alexa Fluor 546-conjugated secondary antibodies (1:200) for 1 h at room temperature. Thereafter, the antibodies were washed in PBS four times for 10 min, and counterstained with Hoechst 33342. The cells were examined on a Zeiss LSM510 confocal scanning microscope. In a single experiment, cells were counted by eyes in the stored images (original magnification, ×40) until at least 100 cells or 100 foci were registered.

### Western blot analysis

The treated cells were lysed with Tween-20 lysis buffer (50 mM HEPES (pH 7.4), 150 mM NaCl, 0.1% Tween-20, 10% glycerol, 2.5 mM EGTA, 1 mM EDTA, 1 mM DTT, and 1 mM PMSF). Equal amounts of protein were analyzed by sodium dodecyl sulfate-polyacrylamide gel electrophoresis (SDS-PAGE). Thereafter, the proteins were transferred to nitrocellulose membranes and analyzed by specific primary antibodies. Proteins were detected with enhanced chemiluminescence after incubation with horseradish peroxidase-conjugated secondary antibodies.

### Statistical analysis

Data were expressed as mean ± SD. Every experiment was repeated at least twice. Statistical analysis was performed using GraphPad Prism software version 5.0 (GraphPad, San Diego, CA, USA). Student’s *t* test and ANOVA were used when appropriate. Differences were considered significant if P < 0.05.

## Competing interests

The authors declare that they have no competing interests.

## Authors’ contributions

LJJ carried out all the experiments and drafted the manuscript. QA, WH and LJN participated in the cell culture. WJJ and ZY participated in the experiment design and drafted the manuscript. All authors read and approved the final manuscript.
